# Influenza Infectious Dose May Explain the High Mortality of the Second and Third Wave of 1918–1919 Influenza Pandemic

**DOI:** 10.1371/journal.pone.0011655

**Published:** 2010-07-26

**Authors:** A. Cristina Paulo, Margarida Correia-Neves, Tiago Domingos, Alberto G. Murta, Jorge Pedrosa

**Affiliations:** 1 Life and Health Sciences Research Institute, School of Health Sciences, Universidade do Minho, Braga, Portugal; 2 Environment and Energy Scientific Area, DEM, and IN+, Center for Innovation Technology and Research, Instituto Superior Técnico, Lisboa, Portugal; 3 Instituto Nacional dos Recursos Biolgicos - IPIMAR, Lisboa, Portugal; University of Oxford, United Kingdom

## Abstract

**Background:**

It is widely accepted that the shift in case-fatality rate between waves during the 1918 influenza pandemic was due to a genetic change in the virus. In animal models, the infectious dose of influenza A virus was associated to the severity of disease which lead us to propose a new hypothesis. We propose that the increase in the case-fatality rate can be explained by the dynamics of disease and by a dose-dependent response mediated by the number of simultaneous contacts a susceptible person has with infectious ones.

**Methods:**

We used a compartment model with seasonality, waning of immunity and a Holling type II function, to model simultaneous contacts between a susceptible person and infectious ones. In the model, infected persons having mild or severe illness depend both on the proportion of infectious persons in the population and on the level of simultaneous contacts between a susceptible and infectious persons. We further allowed for a high or low rate of waning immunity and volunteer isolation at different times of the epidemic.

**Results:**

In all scenarios, case-fatality rate was low during the first wave (Spring) due to a decrease in the effective reproduction number. The case-fatality rate in the second wave (Autumn) depended on the ratio between the number of severe cases to the number of mild cases since, for each 1000 mild infections only 4 deaths occurred whereas for 1000 severe infections there were 20 deaths. A third wave (late Winter) was dependent on the rate for waning immunity or on the introduction of new susceptible persons in the community. If a group of persons became voluntarily isolated and returned to the community some days latter, new waves occurred. For a fixed number of infected persons the overall case-fatality rate decreased as the number of waves increased. This is explained by the lower proportion of infectious individuals in each wave that prevented an increase in the number of severe infections and thus of the case-fatality rate.

**Conclusion:**

The increase on the proportion of infectious persons as a proxy for the increase of the infectious dose a susceptible person is exposed, as the epidemic develops, can explain the shift in case-fatality rate between waves during the 1918 influenza pandemic.

## Introduction

During the 20th century there were three influenza pandemics [1], [2] characterised by the occurrence, within one year of, at least, two to three successive epidemic waves and by an increase in the case-fatality rate in the later waves [3], [4]. The 1918 influenza pandemic caused up to 40 million deaths [1], [5], [6], a number that far exceeded the number of fatalities in the 1957 and 1968 influenza pandemics, of about 2 and 1 million deaths, respectively [7]–[9]. The reasons behind the exceptionally high case-fatality rate in the 1918 influenza pandemic have been associated to the virus pathogenesis [10]–[12], the absence of antibiotics to treat secondary bacteremia infections [13], [14] and to a debilitated health care system, exhausted by a frail population found at the end of World War I [3]. The increase in the case-fatality rate between waves, on the other hand, is attributed to the emergence of a pathogenic virus type after a genetic change in the circulating virus [10] or to a reassortment with a zoonotic influenza virus [15]–[17]. The precise time at which the new virus type emerged is not known and at least two hypothesis have been proposed [1]. Some authors advocate that the virus emerged immediately before the Autumn wave [15], [18], whereas others proposed that the virus had seeded itself earlier in 1916 [19], [20]. Supporting the latter hypothesis is the small time interval of six months between the first and second wave for the new virus to spread worldwide, and the increase in the number of deaths from influenza-like illness in military camps and in small civilian communities during the winters of 1916 and 1917 [21]. Furthermore, the rate of evolution of the neuraminidase and hemagglutinin genes, whose coded proteins are determinant in the entry and exit of the virus in the host cell, suggest a possible emergence in 1915–1916 [19]. The protracted period of almost two years, between seeding of the virus and the emergence of the 1918 influenza pandemic, was explained by the restricted travel during World War I which allowed the virus to maintain itself in small civilian communities and in army camps while increasing in virulence [20], [21]. Later on, demobilisation of troops would have aided the spread of the virus worldwide [20]. However, this may not be the reason for the shift in disease severity, given that demobilisation started after the armistice signed in November 11th, that is, after the deadly second wave had peak in most European countries [3] and in many USA cities [22].

In this paper we explore a new hypothesis for the pattern of increased case-fatality rate during the latest waves of the 1918 influenza pandemic. This hypothesis is based on a dose-dependent response according to which influenza mortality increased when healthy susceptible persons were exposed to a high infectious dose of the 1918 influenza virus. The possibility of a dose-dependent response to explain the increased case-fatality rate during the second wave of the 1918 influenza pandemic has never been put forward. This is particularly surprising given the observation, in the laboratory setting, that only inoculation with a median infectious lethal dose, that is the dose that kill 50% of the animals inoculated, in mice [10], [11] and in cynomolgus macaque model [12], caused extensive oedema and haemorrhagic exudates as reported for patients who succumbed to the 1918 influenza pandemic [12].

In this paper we used mathematical modelling to simulate the dynamics of influenza virus infection in an immunological naïve population, from invasion of the virus until one year later. To model the infectious dose we assumed that, in average, the dose is mediated by the number of simultaneous contacts a susceptible person has with infectious ones. We further distinguished between mild and severe disease by assuming a lower or a higher mortality rate, respectively.

## Results

**Figure 1:**
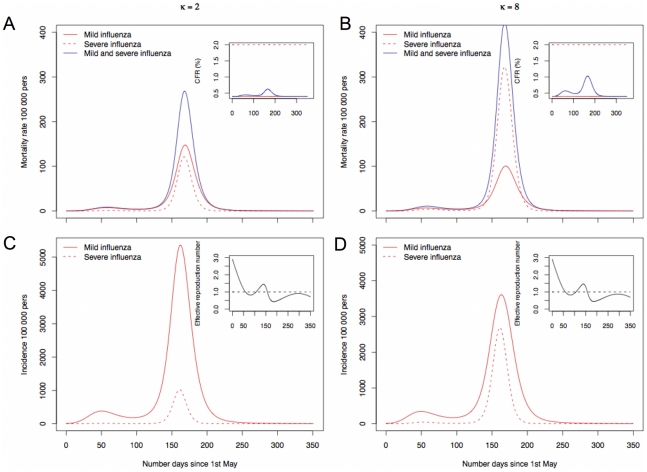
Incidence, mortality and case-fatality rate for influenza pandemic under different levels of multiple contacts between a susceptible person and infectious ones. A and B gives the mortality rate and the case-fatality rate for scenarios 1 (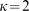) and 2 (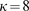). C and D gives the corresponding incidence per 100 000 persons in the population and the effective reproduction rate.

Simulations from the proposed model showed a two-wave pattern and an increase on the case-fatality rate (CFR) during the second wave (Figure 1). This increase results from an increase in the incidence of severe cases that build up as the proportion of infectious persons in the population increases. The CFR for severe disease is 5 times higher then the CFR for mild disease, such that an increase of 1000 mild cases add to mortality 4 deaths whereas 1000 severe cases add to mortality 20 deaths. The increase in the CFR is also higher when the parameter that measures simultaneous contacts is higher, 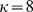 (Figure 1B). During the second wave there is a distinct mortality rate that depends on the infectious dose, here mediated by the number of simultaneous contacts, whereas for the first wave the mortality in both scenarios is almost the same (Figure 1). The first epidemic wave peaked in July, when the effective reproduction number (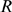) is already decreasing below 1 due to a very low value of the transmission rate (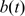) (Figure 1C and 1D). That is, in July the transmission is no longer effective even though there are plenty of susceptible persons. As such, the proportion of infectious persons that build up is not enough to generate many severe cases and the CFR is then maintained near 0.4% during the first wave in both scenarios (Figure 1A and 1B). During the second wave, on the other hand, 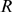 is higher than 1 and the epidemic build up quickly. The second wave peaked in October and the mortality rate is then dependent on the value for simultaneous multiple contacts between a susceptible and infectious persons (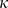).

**Figure 2:**
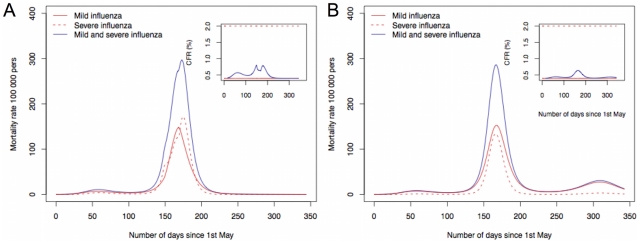
Dynamics of influenza pandemic with varying 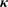 and waning immunity. A a change in the level of multiple contacts as soon as the number of deaths is above 500 from 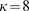 to 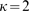 and B a faster rate for waning immunity in a scenario where 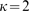.

If the number of simultaneous contacts is decreased from 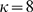 to 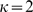 in the middle of the epidemic, there is a decrease in the mortality rate observed (Figure 2A). The total mortality rate among the population when 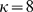 was 4267 deaths per 100 000 persons whereas when 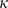 decreased to 2 the total mortality rate decreased to 3773 deaths per 100 000 persons. Decrease in mortality is higher when the change in 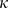 is implemented sooner in the epidemic and has no impact if it is implemented too late.

**Figure 3:**
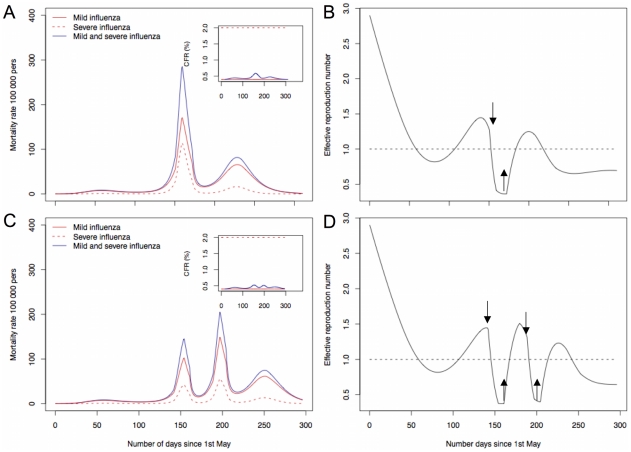
Dynamics of influenza pandemic with volunteer isolation. A and B volunteer isolation started when the number of deaths was above 400 and persons returned to community when the number of deaths was bellow 100 and C and D volunteer isolation started when the number of deaths was above 200 and persons returned to community when the number of deaths was bellow 100. Arrows in B and D indicate the time at which persons leave and return to the susceptible compartment.

In the proposed model a third wave can occur as persons in the recovery compartment wane immunity. Nonetheless, the rate at which immunity is lost has to be high, in the order of 1 year in average (Figure 2B). If the rate is low (table 1), there are only two waves (Figure 2), unless there is introduction of new susceptible persons in the population (Figure 3). If a group of persons became voluntarily isolated, for instance due to the perception of a high number of deaths, returning to the community some days later, several epidemic waves occurred. The number of epidemic waves will depend on the time of the epidemic people leave and return to the community. The lower the value of mortality that alert people leave the community, the higher the number of waves (Figure 3A and 3C). The CFR depends on the proportion of infectious persons in each wave and on 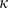. But, for the same transmission rate and population size, the more waves are build up the lower the chance a susceptible person has to make simultaneous contacts with infectious persons in each of the waves and lower the CFR. This is depicted in the simulations. The total number of deaths for 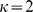 without isolation, was 2984 deaths per 100 000 persons (Figure 1A), whereas with voluntary isolation there were 2947 deaths per 100 000 persons (Figure 3A). If people become isolated at different times during the epidemic, more waves were produced and less deaths occurred. In the scenario producing four waves there were 2884 deaths per 100 000 persons (Figure 3C).

**Table 1:** Parameters used in the model.

Parameter	Value	Reference
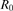 Basic reproduction rate	2–5	[50], [60]
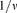 Average period in latent compartment (days)	2	[41]
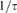 Average period in infectious compartment (days)	5	[41]
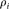 Rate of waning immunity (days)	9.7e-04 (mild), 4.8e-04 (severe)	[51]–[53]
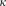 Level of multiple contacts	2 and 8	
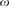 Proportion leaving the susceptible compartment	0.1	
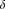 Proportion returning to the susceptible compartment	0.1	
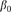	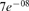	
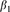	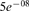	

## Discussion

In this paper we proposed that severe cases resulting from an infection with influenza A virus of a naïve healthy individual is due to a higher infectious dose of the virus. Additionally, we proposed that the infectious dose is mediated by the number of simultaneous contacts established between a susceptible person and infectious ones. In this sense over-crowded places would have been ideal for a susceptible person to be exposed to very high infectious doses of influenza A virus. In 1918 the army camps fit the model by being characterised by a high number of contacts between people and by a high case-fatality rate, sometimes 5 to 8 times higher than the case-fatality rate among civilian communities [23], [24]. This difference in influenza-like illness mortality is sometimes associated to poor conditions in military base hospitals [23] or to a lower lung capacity of soldiers due to the inhalation of gases during the war [20]. However, of note, many of these reports were from training army camps [24] where soldiers had health-care conditions similar to those offered to civilian communities. Differences in the CFR could also result from age related mortality since persons aged between 20–30 years old, similar to the soldiers age range, were the most severely affected during the 1918 influenza pandemic whereas among civilians the CFR might have be muted due to a wider age range. Nonetheless, even between civilian communities, factors such as crowding or continuous exposure, that can be viewed as favouring simultaneous contacts between a susceptible person and infectious ones, were associated to higher mortality rates. Rurality, for instance, was referred as a protective factor for the 1918 influenza pandemic mortality compared to urban areas [25]–[27]. In one of these papers the CFR was estimated and it was found that case-fatality rate was highest in larger towns followed by smaller towns and cities. Villages appeared to yield the lowest case fatality with an estimated 0.96 (0.82 1.09)% and the highest morbidity [27]. This may be indicative that the chance to make simultaneous contacts between a susceptible person and infectious ones is higher in larger cities compared to villages. In villages, contacts are probably easily established between persons, enhancing transmission, but most probably involve, at each time, few infectious persons which diminishes 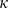 and thus the CFR. We cannot, nonetheless, exclude other factors for the observed difference. Socio-demographic heterogeneity's such as a higher proportion of young people, poorer health and nutrition in urban areas may have also contribute to the difference in mortality between urban and rural areas [27]. More examples that could be indicative of a higher mortality associated to a higher infectious dose include data from two parishes in Norway, where the number of rooms per apartment was associated to a higher mortality during the 1918 influenza pandemic [28], as well as an analysis of mortality data by family in Iceland, that lead the author [29] to propose that the most important determinant of fatal outcome during the 1918 pandemic was associated to greater proximity or repeated exposure to infectious patients, possibly through greater infective dose of the virus, resulting in higher viral burden with “cytokine storm” and death. As in previous examples other factors such as economic level that could determine the nutrition status and access to health care services among persons living in smaller and crowding apartments cannot be excluded [30].

It is generally recognised that there is a minimum infectious dose able to produce infection in naturally occurring influenza in humans. The importance of this dose-dependence is the basis for some of the World Health Organisation recommendations for pandemic influenza interventions. Those interventions are aimed to reduce contacts with infectious individuals avoiding infection of other persons or delaying the spread of the virus and thus prevent disruption of health-care services [31]. The effect of high infectious dose on influenza disease progression, on the other hand, have been shown in experimental animal models [32]–[39]. The dose-dependence is variable, depending on the site of inoculation [35], the host background [40], the host age [38] and the influenza virus type. Overall, a high infectious dose is associated to a higher viral load [33], [34], with a smaller period of time to maximum viral load [33], [34] and with extensive clinical symptoms [32], [34], [39]. In volunteer challenge studies using humans, only the duration of virus shedding was found to be dose-dependent on the intranasal dose whereas the number of symptoms were more dependent on virus shedding [41]. Challenge studies in humans are difficult and results can be confounded by attenuation of the virus, the route of infection and previously acquired immunity [41]. Furthermore, volunteer challenge studies lead to mild or symptomless disease only and may not reflect naturally acquired influenza virus infection characterised by a spectrum of disease states, ranging from clinically symptomless illness through mild infection and to severe, even lethal, viral pneumonia. Ethical limitations in volunteer challenge studies are overcome by the use of mathematical models to reproduce the dynamics of the immune response against an infection with influenza A virus in humans. A robust result from simulations of these models point to an upper threshold on the infectious dose above which the proportion of damaged epithelial cells results in severe influenza disease [42], [43]. For a small infectious dose the disease progresses through an asymptomatic course and for intermediate values of infectious doses the outcome is variable [42] which could, in part, explain the lack of a clear dose-response in human studies.

In our model we assumed that the number of simultaneous contacts between a susceptible person and infectious ones is a proxy for influenza infectious dose. Influenza A virus spreads from person-to-person by droplet transmission [44], aerosol transmission [45] or self-inoculation by contact with fomites or contaminated hands [44], [46]. Both droplet transmission and transmission through contaminated hands needs close contact between susceptible and infectious persons and, although long-range transmission of aerosol particles is possible, the amount of virus sprayed in each sneeze is so small and is so rapidly diluted, as the aerosol disperses, that the risk of infection is probably significant only at the proximity of a susceptible person [44], [45].

In our model we also addressed waning immunity as a possible mechanism to explain a third wave. This mechanism has been previously used to fit a dynamical model to data on the 1918 influenza pandemic and the best fit estimated that the replenish of the susceptible pool due to waning immunity could occur in a time scale from weeks to months [47]. This rate is higher than the one we used in the model but in fact the only difference expected by increasing the rate of waning immunity is a higher morbidity during the third wave and thus a higher CFR, but still lower then the CFR during the second wave. An important aspect not covered by this modelling is the inclusion of asymptomatic cases [47]. If the infectious dose is very low we expect more asymptomatic infectious individuals [42] that in turn will decrease the attack rate, decreasing the number of infectious individuals and thus of severe cases.

Overall, nonetheless, the model reproduces the mechanism we want to show. In fact, according to the model structure case-fatality rate is a non-linear function of the number of infectious individuals, increasing at a higher rate when severe disease cases build-up. This structure differs from other mathematical models [48], [49] where case-fatality rate is a linear function of the number of infectious persons. This difference has important consequences when interpreting historical data on mortality and when considering strategies to mitigate influenza mortality. Case-fatality rate associated to the 1918 influenza pandemic has been estimated as being between 0.3% and 6% [23], [50]. Under this hypothesis, nonetheless, the CFR associated to severe disease has to be much higher than 6% to have in average an observed CFR of 6%. Also, as simulations showed, the number of severe cases, in each wave, decreased when the number of infections was spread along time, which resulted in a decrease of the overall CFR. Adoption of layered non-pharmaceutical interventions, like school closure and public gathering ban, earlier and in a sustained way, have been considered to reduce the attack rate of influenza among persons in the community [31]. However, as simulated by mathematical models, the efficacy of these interventions are greatly dependent on the basic reproduction rate (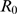) and on the starting time and duration of those interventions [48], [49]. In light of our hypothesis, nonetheless, non-pharmaceutical measures may be more important to reduce case-fatality rates than morbidity. Implementation of such interventions spreads the epidemic into a longer period, decreasing the number of infectious persons at each time in the epidemic, and consequently decreasing the number of severe influenza cases among healthy people and overall mortality.

## Materials and Methods

### Transmission model

**Figure 4:**
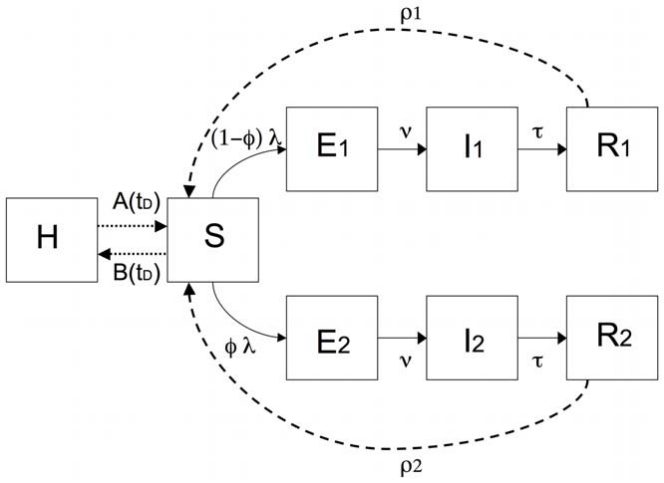
Compartment model for influenza. Each compartment correspond to a class of individuals in the population and arrows indicates transfer of individuals (table 1). In summary S stands for susceptible, E for exposed, I for infectious and R for recovery. The subscript 1 and 2 stands for mild and severe disease. The transfer of individuals from S to H is always 0 except for two scenarios where we assumed that persons after perceiving a higher number of deaths leave the community and become voluntarily isolated. Details on the model are given in the Methods.

To illustrate this hypothesis we used a compartment model to study the spread of the influenza virus in a completely immunological naïve population, from invasion to one year later. (Figure 4). At the start of the simulations all individuals are susceptible to infection (S). After infection, susceptible individuals become exposed (E) for 2 days before becoming infectious (I). The infectious period (I) lasts for 5 days (Table 1) and is followed by the recovery state (R) characterised by resistance to re-infection by an homotypic strain. There are two compartments for exposed, infectious and recovery states corresponding to mild and severe disease. In the model, both disease states are differentiated by the case-fatality rate and by the decay in the antibody titre. In human studies it was observed that after primary infection, antibody titre against an homotypic type decreased with time and three years after maximum antibody response antibody titre was not found in between 11 and 34% of previously infected individuals [51]–[53]. This observation is in agreement with earlier animal experiments were it was observed that the immune response of ferrets following influenza infection, with low or high infectious doses, was resistant to re-infection [32]. Instead, the decay in antibody titre was accelerated for lower infectious doses [32]. Accordingly, in the model, recovery from mild and severe infection was followed by an exponential rate of decay in antibody titre in a way that, respectively, at the end of one or three years, 70% of individuals are still resistant to infection (table 1). The compartmental model is formalised by the following system of ordinary differential equations:
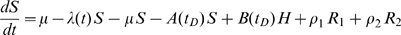
(1)

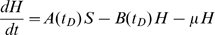
(2)

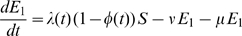
(3)

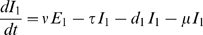
(4)

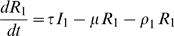
(5)

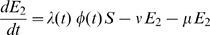
(6)

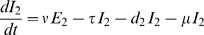
(7)

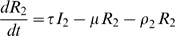
(8)where 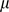 corresponds to the birth and death rate of hosts. The full parameter set is described in table 1. The infection rate 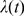 is given by 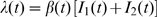. Voluntary isolation, that is the transfer of susceptible persons from S to H depend on the function 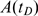 and 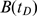 given by;
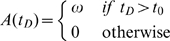


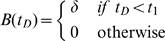
where 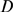 gives the total number of deaths at time 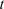.

We used BerKeley Madonna v8.3.12 with autostepsise method to find the numerical results of the model. Initial conditions for the system are given by 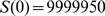, 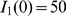 and 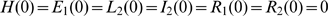. The total population size is 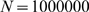.

### Simultaneous contacts

We assumed there is a minimum dose of virus necessary to cause severe infection, and bellow that dose the disease is mild. Severe disease is characterised by a case-fatality rate 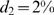 whereas mild disease is characterised by a case-fatality rate of 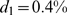.

To model the aggregation of infectious individuals around susceptible persons we used a saturating function of the Holling type II function [54] that is used in population dynamics to model the ability of preys to escape the predator. The Holling type II function is given by
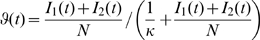
The function 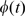 becomes saturated for sufficient large proportion of infectious individuals which was interpreted by a limit in the number of social contacts a susceptible person can established, simultaneously, with infectious persons. This social limit is given by 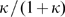. The reason to use this function is as follows. The standard infection rate, 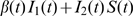, used in epidemiological models, is based on the law of mass action and determines that pairs of individuals interact through chance encounters. This law is only valid for low “concentrations” (e.g., in chemistry, dilute solutions), where simultaneous interactions of three or more individuals have negligible probability. In the context of this work, the relevant interaction of multiple individuals is the simultaneous interaction of a susceptible individual with 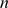 infectious individuals. This is a subset of the pair-wise interactions (i.e., some of the pair-wise interactions are also 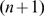 wise interactions). For values of 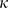 near zero, the social limit is so constrained that there are no interactions of more that two individuals and, even when the number of infectious individuals is very large, all interactions are just pair-wise and there are no severe cases of influenza. At the opposite extreme, when 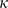 is very large, there is no limit on these interactions, and so, when almost all individuals in the population are infectious, almost all susceptible individuals develop severe influenza.

### Seasonality

Influenza virus activity displays pronounced seasonal cycles in temperate areas with a peak in incidence during winter months. Such seasonal behaviour has been associated with temperature and humidity [55]–[57], to changes in mixing patterns like school terms [57], [58], increased viral production under winter conditions [59] or simply driven by resonance caused by under-detected and small seasonal changes in transmission [59]. We introduced seasonality into the model by assuming that virus transmissibility varies periodically with an yearly cycle. To this end, we modelled the contact rate with a sinusoidal function
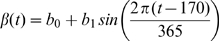
where, the two parameters 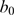 and 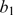 represent the baseline rate of transmission and the amplitude of seasonality, respectively. The function 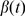 has period of 365 days. Simulations start at 1st May (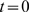) and transmission has a maximum value 260 days after, in 1st January and a minimum value, 75 days after, in 1st July. The values for the 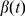 parameters were adjusted such that the seasonally-varying basic reproductive number over an annual cycle summed to 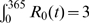 with an amplitude in the range between 1 and 5 [50], [60], [61]. The transmission rate was then scaled according to
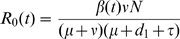
for this model structure the effective reproduction number 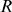, that is the number of cases an infectious individual can generate in a non-susceptible population is given by the 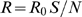.

### Methodological overview

We modelled two scenarios (Figure 2) corresponding to two different values of the parameter 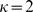 and 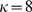, that is the number of simultaneous contacts between a susceptible and infectious persons. A third scenario was simulated by decreasing the value of 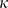 as soon as the total number of deaths increases above 200 (Figure 3A) and a fourth and fifth scenario by allowing voluntary isolation and return to community at two different times in the epidemic (Figure 4). In the fourth scenario persons left the community when the total number of deaths was higher then 200 (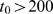) and return when the number of deaths was bellow 100 (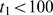), and in the fifth scenario persons left the community when the total number of deaths was above 400 (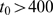) and return when the total number of deaths was bellow 100. The last scenario was simulated by allowing a faster decay in antibody titre (Figure 3B). For severe disease antibody decay was set to 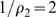 years and for mild disease to 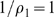 years. Incidence was estimated as the number of infectious persons per 100 000 persons, mortality rate was estimated by the number of deaths by 100 000 persons and case-fatality rate (CFR) was estimated as the proportion of deaths among infectious persons.
